# Intercultural policy in times of crisis: theory and practice in the case of Turin, Italy

**DOI:** 10.1186/s40878-017-0055-1

**Published:** 2017-09-01

**Authors:** Tiziana Caponio, Davide Donatiello

**Affiliations:** 1 Department of Cultures, Politics and Society – University of Turin, and Collegio Carlo Alberto, Moncalieri, Italy; 2 Department of Cultures, Politics and Society – University of Turin, Turin, Italy

**Keywords:** Interculturalism, Policy practices, Immigrant integration, Neighbourhood houses

## Abstract

In this article we analyse how interculturalism, intended as a new paradigm of immigrant integration policies, has been taking shape in an Italian city, i.e. Turin, in the context of the current economic crisis. We argue for the necessity of going beyond official statements on intercultural policy by undertaking a comparative analysis of policy practices in three Neighbourhood Houses (NHs, *Case del quartiere*), which are defined by the Municipality as open and intercultural spaces in which associations and citizens can develop activities aimed at expressing different cultural backgrounds, fostering participation and supporting social inclusion. The study shows how the three NHs pursue different approaches – social, cultural or more political – to interculturalism, somewhat reflecting the social and structural context in which the three NHs were established, as well as problem definitions and policy frames of the founding organisations.

## Introduction

Local immigrant integration policies are currently challenged by multiple crises: along with the economic downturn which has lasted since 2007, the integration crisis (Joppke, 2007) following the terrorist attacks of the mid-2000s in some of the main European cities, and the refugee crisis, with its massive (and mediatised) arrivals, call for profound revisions of cities’ approaches towards migration. So far, the literature has focused primarily on the impact of the ‘integration crisis’, highlighting a mounting backlash against multiculturalism (Vertovec & Wessendorf, 2010) and the emergence of new paradigms such as diversity (Schiller, 2015), interculturalism (Zapata-Barrero, 2016) and mainstreaming (Scholten, Collet, & Petrovic, 2017).

Yet, poorly addressed is the impact of the economic crisis in the definition of these new approaches towards integration (Ambrosini & Boccagni, 2015). Continuous welfare state retrenchments and cuts in public spending might represent important factors leading to the abandonment of costly multicultural programmes or impeding their emergence altogether at the local level. In the case of Italy, immigrant integration policies, which have always been a local affair, have been seriously affected since 2005 by the dramatic cuts of the National Social Policy Fund, which was – and still is – essentially aimed at financing local welfare systems. Therefore, the question arises of how municipalities and local policy-makers have reacted to this unfavourable opportunity structure. In this article, we focus on the emergence of interculturalism as a new paradigm of immigrant integration policies (Zapata-Barrero, 2016) to underscore how it has been concretely framed in an Italian city, i.e. Turin, in the context of the current economic crisis. While explicitly promoted by the Municipality in its official statements, interculturalism is actually defined in very vague terms, which makes it difficult to understand which policy actions and practices should be put in place. To shed light on this puzzle, we argue for the necessity of going beyond official statements and undertaking a comparative analysis of policy practices in three specific places where encounters with diversity and dialogue are supposed to take place, i.e. the so-called Neighbourhood Houses (NHs, *Case del quartiere*).

The article is organised as follows. First, starting from the literature, we discuss the intercultural policy approach in order to highlight its main dimensions and derive hypotheses on the possible consequences of the economic crisis on local immigrant integration policies and on the emergence of the intercultural approach. The second section is devoted to the presentation of the context, i.e. the city of Turin and the NHs analysed, and to elucidate the methodology. The third section presents the results of our research on the three NHs, while in the fourth we attempt to account for the emergence of different variants of interculturalism. In the conclusion we discuss the implications of our study for the literature on local integration policies and identify future research paths.

## Interculturalism: paradigm or buzzword?

The local turn in the study of immigrant integration policies has led to the consolidation of an increasingly articulated body of literature which underscores the relevance of and pays attention to various aspects of local immigrant policies. Whereas some scholars have focused on policy content, following a line of research which started to develop as early as in the mid-1990s (see for instance: Alexander, 2007; Bak Jørgensen, 2012), others have been particularly concerned with local policy-making processes, looking at how immigrant integration policies are decided upon and implemented in local-level policy-making arenas (Caponio, 2010; Caponio, Jubany, & Guel, 2016; Schiller, 2015) or across different levels of government in complex multilevel governance arrangements (Dekker, Emilsson, Krieger, & Scholten, 2015; Scholten, 2013).

As already mentioned above, more recently the literature has also drawn attention to the emergence of new paradigms in local immigrant integration policy, among which interculturalism stands out for its influence on the policy practitioners and scientific debates. Existing definitions of this policy approach (see: Zapata-Barrero, 2016; Salée, 2010; Meer, Modood, & Zapata-Barrero, 2016) usually stress the contrast with the notion of multiculturalism. Hence, according to this literature, interculturalism emphasises individual diversity rather than group difference, interaction and dialogue rather than recognition and separation, and the sharing of what is common rather than exhibiting what is unique.^1^

Yet, what seems to be missing in the debate on interculturalism and on local immigrant integration policies more generally is specific attention to the level of policy practices, i.e. following Lipsky’s (1980) classic study of street-level bureaucracy, on how certain policy philosophies or ideas are concretely implemented and translated into specific actions. This is a crucial step in order to understand the conditions that migrants face in the process of becoming an ‘accepted part’ of the receiving society (Penninx & Martiniello, 2004). Policies cannot just be identified with official programmes: along with rhetorical frames, policies are also the product of ‘action policy frames’ (Schön & Rein, 1994), i.e. the frames that are used in implementation processes in order to construct the problem in a specific situation. Analysing policy practices appears to be of extreme relevance if we are to understand what interculturalism really means, especially in a context of economic crisis and shortage of resources.

The few existing analyses of policy practices highlight how social workers and street-level bureaucrats play a crucial role in the adaptation of social services in a ‘culture-friendly manner’ (see for instance: Vermeulen & Stoijtin, 2010; Boccagni, 2015). However, the practices carried out by street-level bureaucrats do not necessarily reflect a particularly open attitude towards foreign migrants, but can be more prosaically be the result of pragmatic considerations about the difficulty of dealing with immigrants’ cultural backgrounds (Caponio, 2010). Such difficulties are likely to be further exacerbated in times of austerity and budget cuts, which will impose a reduction in the available services and/or a stricter selection of target groups. The link between economic crisis and policy practices, while intuitive in many respects, has not been the object of specific attention in the migration policy literature. In this article we intend to elucidate this link by looking at how the emerging intercultural policy paradigm, explicitly adopted by the city of Turin in the mid 2000s, has been concretely translated into policy actions and implementation practices since 2007.

The emerging literature on interculturalism identifies three dimensions or normative drivers of this policy approach: political, social and cultural-normative drivers. Ideally, the intercultural policy approach should be aimed at pursuing a balanced and comprehensive framework where these three different views coexist and reinforce each other (Zapata-Barrero, 2016). The political driver is at the basis of a contractual theory of interculturalism (Bouchard, 2012), which posits as fundamental the reaching of a dynamic equilibrium between the goal of ensuring the survival of the national identity and, on the other hand, that of respecting the rights of ethnic minorities. The social driver emphasises the cohesion theory, which regards intercultural policy as a way to overcome social conflict and segregation due to the lack of communication between different expressions of diversity in the society (Cantle, 2008). The cultural driver is at the basis of a ‘constructivist’ (Zapata-Barrero, 2016) or cultural approach, that interprets interculturalism as an instrument to promote the cultural capabilities of individuals and positive interaction among people with different cultural backgrounds, which should lead to the emergence of a new, creativity-based, diverse society.

Existing studies on intercultural policies show how these three drivers usually underlie the strategic goals and official statements of most of the cities that in Europe and beyond explicitly adhere to the intercultural approach (Lüken-Klaßen & Heckmann, 2010; Rocher, 2015). However, the empirical question remains of how these three drivers are translated into concrete policy actions and which measures are actually prioritized by policy-makers who are in charge of implementing intercultural policies in a specific context.

Considering Zapata-Barrero’s (2016) drivers of interculturalism, we can suppose that in a context of economic crisis we should expect a particular emphasis on the social dimension, in order to avoid conflict and marginalisation while enhancing social cohesion and equal access to resources on the territory. In a context of reduced economic opportunities, the political dimension may also become of increasing relevance, because of the necessity to prevent divisions between national majority and ethnic minorities, to allow for mutual understanding while avoiding radicalisation and violence (Lüken-Klaßen & Heckmann, 2010).

Yet, an alternative hypothesis can also be put forward. In the context of the current economic crisis, addressing the political and, even more, the social dimensions of interculturalism would impose further costs on already meagerpublic budgets. This is likely to be the case for instance, on the political dimension, of subsidies for immigrants’ associations, or, on the social one, of specific assistance for disadvantaged families, immigrant and native alike. The cultural dimension on the other hand, can appear less demanding in this respect and can even be translated into low-cost policies like providing a public room at no cost or granting permission for the use of a park to organise intercultural events or festivals while requiring at the same time that the organisers will take charge of security and cleaning up. Hence, by analysing policy actions undertaken at a grassroots level, we aim to understand whether interculturalism is really a new paradigm in local immigrant integration policy, as existing scholarly literature seems to assume, or rather if it is just a new a buzzword, used by policy-makers to seemingly reinvent integration policy in times of crisis.

## Context and methodology

To assess the hypotheses presented above, in this article we analyse intercultural policy practices in the city of Turin at a district or neighbourhood level. This level of analysis appears of crucial relevance both from a theoretical and factual point of view. From the theoretical standpoint, since, following Zapata-Barrero (2016), the very essence of interculturalism lies in social interaction, the neighbourhood level appears to be a more appropriate site for analytical observation; from a factual perspective, on the other hand, the intercultural approach promoted by the Municipality of Turin since the mid-2000s has emphasised the strategic relevance of neighbourhoods as contexts where everyday interactions between immigrants and national residents take place (Caponio & Ricucci, 2015).

Although the goal of this article is not to assess how effectively the city’s official policy goals and views on interculturalism have been translated into concrete practices, here below we first provide a brief account of the city’s intercultural approach to immigrant integration with particular attention to the interventions targeting neighbourhoods. Hence, we describe the research metholodogy and the operationalisation of the three dimensions of interculturalism discussed above.

### The context. The city of Turin’s intercultural policy

The intercultural approach to immigrant integration of the Municipality of Turin dates back to 2006, when the Department for Integration was created with the mandate of defining a coherent intercultural policy.^2^ The policy action of this Department developed along four main axes of intervention: 1) intercultural education projects for young children and second generations; 2) conflict mediation in the use of public space and access to public services; 3) immigrant associations’ civic engagement; 4) intercultural dialogue and appreciation of diversity at a district level. As pointed out by various research studies (Caponio & Ricucci, 2015), the social inclusion dimension of interculturalism clearly has always featured as crucial, since both intercultural education and conflict mediation were conceived as interventions aimed at avoiding social marginalisation and contrasting the formation of disadvantaged groups. However, the specific attention devoted to the strengthening of immigrant associations on the one hand and to the promotion of cultural diversity on the other testify also to the relevance of the political and cultural dimension respectively.

Regarding this last aspect, since 2007 the Municipality has supported the creation of specific spaces, the so-called Neighbourhood Houses (NHs), which were thought of as providing a framework for promoting intercultural encounters and an appreciation of diversity. In fact, the official *manifesto* of the NHs’ Network describes them as ‘places to accommodate, through *intercultural activities*, all citizens, from children to the elderly, without discrimination by gender, nationality, social background and religious belief’ (Casa del Quartiere di Torino, 2012). However, consistent with the city’s more general intercultural approach described above, the social dimension of interculturalism is also explicitly mentioned, since the NHs are supposed, especially in the context of the economic crisis, to provide opportunities in terms of sociability and creation of new social ties to ‘find collective responses to common needs’ (Casa del Quartiere di Torino, 2012).

First projects and feasibility studies of NHs began in the early 2000s on the initiative of various civil society associations and NGOs and with the support of the Municipality The first House to be officially opened was *Cascina Roccafranca,* inaugurated in 2007 in the Mirafiori Nord neighbourhood, an area that was undergoing a process of urban regeneration thanks to the EU-funded programme *Urban II*. Since then, eight other NHs^3^ were founded in different neighbourhoods. Each one was the result of a unique and independent bottom-up process (Roman, 2014). The Municipality offered logistical and financial support to these initiatives, therefore contributing to their institutionalisation but never interfering with their activities.Only in 2012 did the Municipality attempt to connect the different houses in a Network (*Di Casa in Casa*), with the goal of rationalising and streamlining the funding system. In fact, in 2012, to face the continuous cuts to the National Social Fund, and therefore the decreasing availability of financial resources at the local level, the Compagnia di San Paolo, a private banking foundation based in Turin which has historically played a crucial role in sustaining local development and social policy, signed an agreement with the Municipality for the ‘Development of Welfare Programmes’ (2012–2013),^4^ which included financial support to NHs. Whereas before each house applied for Compagnia’s grants autonomously, after the signing of the agreement the Integration Policy and Urban Regeneration Department receives a yearly budget of about 500,000 euros for developing the NHs’ activities. The share assigned to each NH is established on a case-by-case basis and in any case does not impose any obligation in terms of activities to be carried out, which are still decided autonomously by each NH. In any case, NHs are explicitly requested to develop the ability to attract external resources and to find alternative funds.

### Methodology

For this study, we consider three NHs. First we have selected the two NHs located in the areas that in 2014 had the highest ratios of immigrant residents^5^: *Bagni Pubblici via Agliè* in the Barriera di Milano neighbourhood, where the percentage of foreigners among the total resident population is 23.2%, and *Cecchi Point - Hub Multiculturale* in the Aurora neighbourhood, where the ratio is 21.4%. The third selected NH, i.e. *Casa del Quartiere di San Salvario*, is in the San Salvario neighbourhood, an area which has historically hosted different waves of migrants, from Southern Italians in the 1960s to first foreigners in the 1970s and 1980s, even though currently the ratio of foreign residents is 13%, below the city average of 15%.

To better understand how NHs have interpreted interculturalism in the context of the recent economic crisis, we have carried out three qualitative case studies on each of the selected NHs including: analysis of official documents (NH websites, monthly planning of their activities, lists of the associations involved); ten in-depth interviews (conducted between June 2015 and February 2016) with the directors/mangers of the three NHs, presidents/founders of some associations actively involved in the NHs, the coordinator of the Network *Di Casa in Casa,* the deputy Mayor for Urban Renovation and Integration at the Municipality of Turin; and observant participation in some specific intercultural events (exhibitions, meetings, celebrations, festivals, etc.). Our fieldwork was aimed at gathering information on intercultural practices in each of the three selected NHs. Below we provide details on the operationalisation of the three dimensions of interculturalism identified by Zapata-Barrero (2016) in terms of expected policy practices.

Regarding the *social dimension* of interculturalism, we should expect to find initiatives targeting immigrants and natives alike which are aimed not only at reducing marginalisation, poverty and social inequalities more generally, but also at fostering a sense of inclusion in the local community. This is the case in the instance of social services offered to migrants and people in need, in terms of community welfare for the neighbourhood and as mechanisms for strengthening local solidarity, proximity relations and social cohesion.

On the *cultural dimension* of interculturalism we consider activities aimed at promoting cultural capabilities, supporting positive interaction and transforming potential conflict into opportunities for socialisation and mutually beneficial contacts. In more concrete terms, we have looked at how common spaces – inside or outside the NHs – have been used to organise intercultural events and expressive activities supporting a symbolic understanding of other cultures and the emergence of new common cognitive frameworks based on the added value of diversity.

Last but not least, on the *political dimension* of interculturalism we report as particularly significant those participative solutions aimed at favouring immigrants’ involvement in the NHs decision-making processes and at preventing divisions, such as the – not occasional – inclusion of migrant representatives and associations in steering committees or coordination assemblies, their participation in the everyday running of the NH, as well as initiatives aimed at promoting and strengthening the dialogue among the beneficiaries of NH services.

As is clear, the allocation of policy actions to each dimension should be understood as a purely analytical exercise. We are aware that specific policy actions can have multiple and often overlapping purposes, while the boundaries among the three dimensions of interculturalism may appear less clear once confronted with specific practices. However, the operationalisation of these three dimensions can help to identify the action frames underlying policy-makers’ practices, and therefore the different understanding of interculturalism which would otherwise remain unspoken.

## The NHs. Three case studies

### Casa del Quartiere di san Salvario

This NH was opened in 2010 and is located in a densely populated residential area – San Salvario – that in the second half of the 1990s underwent a phase of social crisis due to the rapid increase of foreigners, the degradation of housing conditions and rising conflicts with the native resident population (Allasino, Bobbio, & Neri, 2000).

In 2003, to face these challenges the Municipality, in the context of a more general strategy of urban renewal of disadvantaged neighbourhoods, promoted the establishment of the Agency for the Local Development of San Salvario,^6^ an organisation composed of various associations, social cooperatives, NGOs, citizens committees and trade unions. The Agency worked to improve the quality of life in the neighbourhood with interventions on housing conditions and social disadvantage, in addition to the promotion of cultural events. In 2005, the Agency took part in a call for urban regeneration projects launched by the Vodafone Foundation. The proposed project, which was actually aimed at setting up the NH, was selected and more than 400,000 euros were allocated for the restoration of a municipal building located in the neighbourhood, the former public baths of Via Morgari – 650 sq. m interior structure and 470 sq. m of courtyard – closed in 2002. The renovation works lasted until 2010.

As highlighted by the Agency’s Director, work on the renovation of the building started "in a context already marked by a shortage of resources… we had sensed the crisis of local government finances, and the House was thought to have a high level of self-financing". To this end, the NH was equipped with an interior restaurant-café called “Municipal Baths”. This is outsourced every two years to a cooperative selected through a public call and its revenues contribute to about 75% of the annual self-financing share. Since 2013 a contribution of 80,000 euros per year (40% of the budget) is allocated to the House on the basis of the agreement between the Municipality and the Compagnia di San Paolo (see above), while since 2011 the District Council (Circoscrizione 8) also began to assign limited funding to the Agency which gradually decreased from 20,000 to 5000 euros per year. In addition, associations and groups that use the NH rooms and spaces for their activities are asked to make a small contribution, as is the case with individuals who choose the NH to organise celebrations and private parties.

The Agency is responsible for managing the NH and provides organisational support to those groups or individuals who wish to carry out their initiatives there. The Agency never adopted a programme of activities or specific guidelines: the goal is to gather together different kinds of actors – associations, informal groups or individuals – in a common space, giving them the opportunity to meet and establish new contacts, therefore promoting practices of togetherness in an original and innovative way. In this context, specific attention has always been paid to migrants’ integration, precisely because one of the main goals of the NH was to "show that foreigners could be a key element for the renovation of the neighbourhood" (Roman, 2014, p. 18).

It would seem, therefore, that in the San Salvario NH interculturalism has been translated into practices inspired especially by a cultural approach. In fact, compared to cultural initiatives, the investment in the social dimension of interculturalism has been far less pronounced. As of today, the NH has a help desk providing information and assistance to refugees and asylum seekers run by the Mosaico association; a legal counselling service offered by experts in migration laws; a series of regular meetings organised by the delegates of the Filipino and Senegalese Consulates of Milan to renew passports and provide other administrative procedures; Italian language courses; weekly after school activities and the Children’s House recreation centre on Saturday carried out by a Catholic association active in the neighbourhood. This last initiative is open to all children although the majority of those attending are foreigners.

On the other hand, the NH promotes many cultural activities aimed at fostering interaction between immigrants and the local community. Several immigrant associations have their headquarters in the NH (in particular Senegalese, Moroccans, Filipinos, Peruvians), and they carry out regular activities as well as occasional events, such as the celebration of cultural traditions or religious festivities like Ramadan. Some of these events, e.g. exhibitions, book presentations, book-sharing, solidarity cocktails, dinner and ethnic cooking classes, workshops, music and dance performances, conferences etc., attract a mixed turnout of Italians and foreigners.

Concerning the political dimension, the participation of immigrant associations in running the NH seems to have been more relevant in the initial phase. At the time of our investigation, there were no immigrant associations among the member organisations of the Agency. In the Director’s opinion this appears to be linked to the low number of associations permanently operating in the San Salvario district. However, the scarce representation of immigrants in the governance of the NH may also be the result of the multiple changes undergone by the neighbourhood. Compared with the 1990s and early 2000s, the presence of first migrant men and of migrant families has considerably reduced because of the gentrification process that the area underwent throughout the 2000s. The real estate market has become less and less accessible to foreign migrants, whereas today the area attracts primarly middle-class native families and young people.

To sum up, San Salvario NH was originally set up in a context of scarce financial resources and was conceived of from the beginning as reliant upon a high level of self-financing. The gradual reduction of funds which occurred over time – as shown by the decreasing funding assigned by the District Council – has not led to any revision of the NH’s intercultural approach since the cultural dimension continues to be preferred. The continuity seems to primarily reflect the changes undergone by San Salvario in recent years, from an immigrant neighbourhood to a culturally dynamic middle-class area populated by young to middle aged people (Bolzoni, 2015).

### Cecchi point – Hub Multiculturale

The explicit reference in the name of this NH to multiculturalism is indicative of the relevance of the immigrant population in the Aurora neighbourhood. Cecchi Point started its activity as early as 2001 but only officially became part of the NHs network in 2011. At the beginning it was established as a centre for young people by the association *Il Campanile Onlus*, that was particularly involved in educational and training activities for the adolescents, including immigrants, of the Porta Palazzo area.^7^ Thanks to an agreement with the District Council (Circoscrizione 7), Cecchi Point could take advantage of a broad abandoned area (2500 sq. m). In 2009, a renovation project was promoted by the Municipality and developed with the financial support of the Vodafone, Umanamente and Compagnia di San Paolo foundations, at an overall cost of 1.2 million euros. The intention was that of transforming the centre in something similar to *San Salvario*. With the restructuring and the recovery of further spaces other associations have joined Cecchi Point which has become a centre open seven days a week, to people of any age and nationality willing to propose activities or to use the available spaces. The initial renovation led to the opening of a building destined for education activities for children and young people, with a multi-purpose hall, and on the business side with the “Cecchi mangia” restaurant-café and offices. More abandoned premises were later renovated, i.e. the creative workshop area (carpentry and tailoring), the dance room, the gym and the underground theatre.

Il Campanile is the only association responsible for the running of the NH and its current staff consists of nine people: two educators; an administrative clerk; and six waiters and cooks employed by the restaurant. For the planning of the NH activities, Il Campanile set up a board including four representatives of other associations. To date, the NH activities have been focused on three areas: socio-educational (the main area due to high demand in Aurora for services), artistic events and creative workshops.

The economic crisis has affected the NH significantly, as reported by an interviewee who sadly said: "We were born in the wrong time". Between 2011 and 2013, Cecchi Point received 100,000 euros from the Municipality per year, with a promise that a further 40,000 euros would be granted from 2013 onward for the running of the daytime youth centre. But the annual budget has been actually reduced in the following period: on the basis of the agreement with Compagnia di San Paolo, Cecchi Point received 80,000 euros (the same amount as San Salvario NH), plus the expected 40,000 euros of Municipal funding for the daytime youth centre. This budget cut led to the reduction of educators – from five to three – in the latest years. Additional revenues to the municipal funding are guaranteed by the restaurant-bar and by the renting of spaces for ad hoc events.

On the side of interculturalism, considering the ongoing practices, Cecchi Point carries out actions which are primarily of a social type. First, a help desk providing counselling and assistance for migrants is open. But, as anticipated, the core activity is the educational one, which in fact involves many foreign children: Aurora is a neighbourhood where there is a high demand coming from immigrant families for educational support, to the extent that foreigners (especially Egyptians and Nigerians) represent around 80% of children attending after-school activities. These families, though, do not seem interested in the other initiatives developed by the NH, especially in the artistic and cultural field. Some immigrants attend the courses offered by the workshops’ labs, especially if they are offered for free or with a symbolic fee. Furthermore, the restaurant-café is regarded as an employment opportunity for migrants: currently, a young Albanian man works as a waiter and the assistant cook – from Bangladesh – has received a work grant from the Municipality, while the previous main chef was a young Moroccan.

As is clear, in the case of Cecchi Point the involvement of foreigners in initiatives encouraging interaction with Italians is difficult. In fact, the cultural dimension of interculturalism appears to be poorly developed. The only activities of this kind are those carried out by Video Community, an association implementing social communication projects aimed at creating a dialogue between people with different social and cultural backgrounds; and the theatre performances that usually involve immigrants but do not seem to attract as spectators the neighbourhood’s foreign residents. Furthermore, the associations active in this House are very few, and usually they just rent spaces for celebrating events attended almost exclusively by compatriots, such as the prayer nights, the Chinese New Year’s Eve and the Senegalese celebration of May (the only event also able to attract Italians). Likewise the political dimension of interculturalism does not seem to be relevant in the practices carried out by this House. We have not found any type of immigrant participation in internal decision-making processes, either at the planning stage or in the current steering committee.

Unlike the previous NH, Cecchi Point has undergone a significant reduction in its budget since 2013, when the annual allocation granted by the Municipality was lower than initially established. Consequently, a clear effect of the crisis has been the reduction in the number of educators working in the NH’s staff. But, despite these cuts, a social understanding of the intercultural policy was maintained, with core educational activities matching the increasing demand coming from Aurora’s immigrant families. In other words, no revision of the original mission of the NH has occurred.

### Bagni Pubblici via Agliè

This House is located in the historical Public Baths building of the Barriera di Milano neighbourhood (Circoscrizione 6). It is the smallest NH, since it does not have a backyard. The Public Baths were re-opened by the Municipality in 2006, and today this service is still in place. Cultural, social and socialisation activities began only later in 2007 when a consortium of social cooperatives, Kairos Consortium, responded to the “Immigrants: new citizens” call of Compagnia di San Paolo, and started the project – with the mediation of Municipality – of becoming an NH. Since the beginning the initiatives were explicitly addressed to immigrants, even though the NH was conceived as a centre open to everybody. Barriera di Milano is an high-density immigrant neighbourhood, yet in this area there were no public spaces promoting intercultural exchanges and integration practices. Compagnia allocated a grant of 60,000 euros and the Kairos Consortium was entrusted by the Municipality with the running of the Public Baths for a ten-year period (2009–2018). Furthermore, in order to undertake the renovation work, Kairos Consortium received specific funding in the context of the Urban III project and took out also a mortgage.^8^

The original aspect of the Kairos Consortium proposal was its aim to involve resident citizens as much as possible in the concrete definition of the activities to be carried out by the NH. Such a strategy appears to have been successful, since when the funding of Compagnia ended in 2009 there were still activities ongoing as well as a high level of citizens’ participation. Therefore, Compagnia decided to renew the fund until 2013, when the Network of NHs was established. Today, funding is ensured by an agreement between Compagnia and the Municipality: as *Bagni Municipali* is the smallest structure, they receive 40,000 euros per year, which is exactly half of the other NHs analysed.

Regarding the sources of funding, this NH is penalized by the fact that it does not have spaces available to rent to associations and private individuals. However, the house has an internal commercial bistro (“Acqua Alta”) that is run by the managing board. From the point of view of internal governance, there is only one appointed person – the Director, employed by the Kairos Consortium – and the management model is based on sharing and informality. Twice a year there is a meeting between the Director, a representative of the District Council (Circoscrizione 6) and representatives from various associations that can join the meeting voluntarily. Despite there being no formal steering committee in place, immigrants are involved in decision-making processes through their daily participation in the life of the House and thanks to their inclusion in ad hoc projects. As a Senegalese tailor, founder of a workshop offering sewing courses (“Baobab Couture”) told us, "we are a permanent coordination assembly". Currently the staff is composed of nine persons: the Director, four (one foreign) employees in the bistro, while the showers service is run by two Italians and two immigrants.

As mentioned above, *Bagni Pubblici via Agliè* was founded with the explicit goal of promoting interculturalism and immigrant integration. With regard to the social dimension, the House offers basic public services to people in need, foreigners and Italians alike. On the basis of estimates done by the Director, the showers have around 60 users per day on average, with a prevalence of foreigners (80%). The effect of the economic crisis is clearly reflected in the increased use of the showers (which has reached a peak of 90 per day) and in the activity of the help desk service, offering information and assistance on job applications, support schemes, and social cards (currently open four days a week instead of two as in the past). In addition, as in the case of *Cecchi Point*, this NH internal bistro is also regarded as an employment opportunity for immigrants. Other examples of initiatives aimed at fostering social cohesion are, on one hand, various workshops (tailoring, photography, silkscreen printing, ceramics) and, on the other, language course, both in Arabic and Italian.

But this House has also developed many cultural activities related to the theme of interculturalism. A small art gallery hosts temporary exhibitions: in particular, the project “A dive in Barriera” (“Un tuffo in Barriera”) supports foreign artists who arrived in Turin as immigrants and who, due to the need to find a job, had to stop their artistic activity. The idea is to give artists the opportunity to express their own talent by showing Italian people that being in a multicultural context can be mutually enriching "even without living in New York". Another important project aimed at generating positive interaction between immigrants and the receiving community is “Drops of writing” (“Gocce di scrittura”), a series of public readings of books written in Italian by immigrants from different countries. Furthermore, in the House there are various ethnic associations that regularly perform activities: Malian, Ghanaian, Senegalese and Moroccan Associations. Furthermore, the House hosts debates (about such issues as discrimination, diversity, and religious dialogue) and ethno-music festivals.

Taking into account the political dimension of interculturalism, in this House immigrants appear to be deeply involved in everyday decision-making. This is due in part to the informal model of governance and in part to the specific way residents (especially foreigners) have been included, i.e. as highlighted above, through their active participation in the NH activities.

To sum up, compared with the other two NHs analysed in this article, *Bagni Pubblici via Agliè* has always received a small budget, yet this shortage of resources seems to have been countered by the bottom-up participation of the people and associations of the neighbourhood, leading to the development of a comprehensive approach to interculturalism. Our interviewees point out how in recent years there has been an increase in the demand for some basic services such as the showers and the help desk, therefore requiring an intensified commitment to social activities. Nevertheless, cultural initiatives remain lively.

## Comparative discussion

Table 1 summarises the main findings of our case studies. As is clear, three different approaches to interculturalism emerge. In the case of *Cecchi Point* the social dimension prevails, as emphasised by the fact that this NH carries out various services targeting disadvantaged people living in the area in general and immigrants more specifically (Italian language courses). The other two cases instead come closer to a more comprehensive approach to interculturalism, i.e. one in which, as emphasised by Zapata-Barrero (2016), the three different constitutive dimensions coexist and reinforce each other. However, *Casa del Quartiere San Salvario* seems to favour the cultural dimension, whereas *Bagni Pubblici via Agliè* shows a wider range of activities spanning the three dimensions.

**Table 1:** Interculturalism dimensions and practices in the three NHs

Interculturalism Dimensions	Interculturalism Practices	*Casa del Quartiere* *San Salvario*	*Cecchi Point* *Hub Multiculturale*	*Bagni Pubblici via Agliè*
Social	Help desk or counselling service	+	+	+
	After-school activity	+	+	−
	Basic public services (i.e. showers)	−	−	+
	Employment at interior restaurant-cafè	−	+	+
	Italian language course	+	+	+
	Workshops and labs	−	+	+
Cultural	Intercultural events	+	−	+
	Exhibitions or book launches	+	−	+
	Expressive and symbolic activities	+	−	+
	Presence of immigrant associations	+	−	+
Political	Participation at the preparatory stage	+	−	+
	Informal involvement in decision-making	−	−	+
	Inclusion in steering committee	−	−	−

The differences among the three NHs are to some extent the result of the bottom-up process which led to their initial set up and gradual institutionalisation. In fact, the Municipality never established NHs from above but just promoted the conversion into NHs of some pre-existing structures, as observed in the case of *Cecchi Point* and *Bagni Publici via Agliè*, and allocated funding to this end, without imposing any specific model of intervention. Likewise, the set-up of the NHs Network never implied the adoption of specific measures or practices, under either the organisational or the policy content profile. As a consequence, different understandings and variants of interculturalism emerged, basically reflecting the social and structural context in which the three NHs were established, as well as the policy priorities and the policy frames of the founding NGOs.

With regard to the social context, the characteristics of the neighbourhoods where the houses are embedded in terms of incidence of the immigrant population, type of demand for services, presence of immigrant associations, availability of other socialisation centres etc., seems to have had considerable influence on the development of their activities, as shown in particular by the *San Salvario* NH on the one hand and *Cecchi Point* and *Bagni Pubblici via Agliè* on the other. In the first case, the prevailing cultural dimension reflects a context which has undergone considerable changes over the course of time, attracting more and more young middle-class people while expelling most of the previous migrant residents. The other two NHs on the contrary, are located in popular and still non-gentrified areas, where the presence of immigrants is particularly relevant.

On the other hand, from the structural point of view, the type of activities carried out seems to have been affected by the physical features of the structures, both in terms of the dimensions of the available spaces and of the original use they were intended for. *Cecchi Point* and *San Salvario* for instance can take advantage of premises with enough space to allow the renting of rooms to private individuals and associations. Via *Aglié* on the other hand, has a much more limited space and part of it was already destined to the Public Baths services, which of course attracts a different audience than the one attending *San Salvario* for instance.

Last but not least, it is also important to consider the strategies of the actors involved in founding and running the NHs. *Cecchi Point* for instance has always been managed in a top-down manner by an association working essentially on youth training and educational programmes, and it does not come as a surprise that these kinds of initiatives are still the core activities for this NH. On the other hand, from the beginning, the association Kairos Consortium, which runs *Bagni Pubblici via Aglié*, has adopted a very different definition of the problem, emphasising the need to stimulate the active involvement and participation of individual residents and their associations in the neighbourhood life, including immigrant associations. Whereas in the first case the political dimension is practically absent, in the second one immigrant associations have been able to get involved in the everyday running of the NH, albeit on a very informal basis, since no steering committee or permanent board has been established. In the case of *Casa del Quartiere San Salvario*, the Agency for the Local Development is responsible for managing the House and has always been open to the participation of foreigners – that was one of the main goals of the NH project – but nevertheless within the Agency, as described above, the participation of immigrant associations seems to have run dry over time.

Returning to the hypotheses put forward in section 2, our study shows that the economic crisis does not seem to have affected the three NHs’ intercultural practices in a significant manner. In a context of reduced financial resources, we would have expected either a re-focusing of the activities towards the social dimension, in response to the increasing demand for social services, or a pivot towards less costly cultural initiatives. On the contrary, our results highlight a high level of continuity in the NHs’ original practices, which seems to reflect the specific neighbourhoods’ social contexts, structural features and problem frames of the organisations which founded each NH.

## Conclusion

From the analysis carried out in this article, at least three frames of interculturalism emerge: as a social cohesion policy in the case of Cecchi Point; as a policy for developping cultural innovation in San Salvario; and as a more general approach to shaping interactions between native residents and immigrants on the social, cultural and also political dimension in the *Bagni Pubblici via Agliè*. Rather than representing a new paradigm or a mere buzzword, interculturalism appears to be a malleable policy approach, which can pursue some specific goals, such as interaction with diversity and dialogue, on different planes, i.e. on the social, cultural and/or political dimension.

The economic crisis does not seem to have pushed any of these dimensions to the fore. In fact, our study highlights a certain capacity of resilience at the very local level, i.e. the neighbourhood, where the initiatives were started and put in place by actors, primarily NGOs, whose activities were already strongly embedded in the surrounding social and cultural contexts. From this perspective, it emerges that, in order to understand integration policy paradigms, as well as the way they change over time in reaction to external challenges such as the economic crisis, the Municipalities’ public philosophies or general policy frames are less relevant than the ‘policy action’ frames, i.e. the frames shaped by the everyday mobilisation of different actors around specific initiatives and measures. Promoting social interaction and intercultural dialogue may mean many things to policy-makers: it is only by unraveling everyday grassroots activities that we can figure out how interculturalism can become a concrete policy approach.

For these reasons analysis at the neighbourhood level, so far quite neglected by migration policy scholars, appears to be of the utmost importance if we want to account for immigrant integration policy paradigms. Of course we are aware of the limitations of our analysis in terms of generalisability, since exploring the neighbourhood level does indeed imply a high degree of in-depth knowledge of the contexts. Yet, comparison across neighbourhoods, as well as of a cross-city/cross-national kind, appears of the utmost relevance in order to provide a fresh outlook on the local dimension of migration policy and policy-making, especially by illuminating the interplay between public policy, private actors and civil society, too often obscured in more institutionalist, municipality-based, analyses.

## Footnotes

1 The notion of multiculturalism is indeed much more complex and still very much debated (for a recent contribution see: Uberoi & Modood, 2015). Here we just report the the view of those scholars who have used the concept of multiculturalism in order to single out by contrast the characterising features of interculturalism.

2 Proceedings of the Communal Council, July 10th, 2006, no. 05033/002.

3 At the moment the NHs are: Bagni Pubblici via Agliè (*Circoscrizione –* District 6), Barrito (*Circoscrizione 9*), Bossoli 83 (*Circoscrizione 9*), Casa del Quartiere di San Salvario (*Circoscrizione 8*), Casa del Quartiere Le Vallette (*Circoscrizione 5*), Casa nel parco (*Circoscrizione 10*), Cascina Roccafranca (*Circoscrizione 2*), Cecchi Point-Hub Multiculturale (*Circoscrizione 7*), +Spazio4 (*Circoscrizione 4*) (http://www.casedelquartieretorino.org/).

4 This Protocol between the Municipality and *Compagnia di San Paolo* follows a previous agreement signed in 2007, focused on initiatives concerning immigration, integration and interculturalism. Afterwards, the 2012–2013 Protocol was renewed for another three years in 2014.

5 Source: Turin Population Registry Office, Statistics and Toponomastic Service. Data update on 31 December 2015.

6 The city of Turin in the early years has supported the Agency with an annual contribution of 120,000 euros. For more information on the Agency see the following link: http://www.sansalvario.org/.

7 At the time, the Cecchi Point in the neighbourhood was known as “Maruega Point”: *maruega*, in suburban slang, is a derogatory expression used to refer to Moroccan-born immigrants.

8 For further details: http://www.comune.torino.it/urbanbarriera/.

## References

[CR1] Alexander M (2007). Cities and labour immigration.

[CR2] Allasino E, Bobbio L, Neri S (2000). Crisi urbane: che cosa succede dopo? Le politiche per la gestione della conflittualità legata all’immigrazione [Urban crises. What happens afterwards? Policies aimed at managing migration-related conflicts]. Polis.

[CR3] Ambrosini M, Boccagni P (2015). Urban multiculturalism beyond the “backlash”. New discourses and different practices in immigrant policies across European cities. Journal of Intercultural Studies.

[CR4] Bak Jørgensen M (2012). The diverging logics of integration policy making at national and City level. International Migration Review.

[CR5] Boccagni P (2015). (Super)diversity and the migration–social work nexus: a new lens on the field of access and inclusion?. Ethnic and Racial Studies.

[CR6] Bolzoni, M. (2015, August). *Upscaling diversity? Some reflections on commodification and control of diversity in a trendy multicultural neighbourhood*. Paper presented at the RC21 International Conference on “The Ideal City: between myth and reality. Representations, policies, contradictions and challenges for tomorrow’s urban life”, Urbino, Italy

[CR7] Bouchard G (2012). L’interculturalisme. Un point de vue québécois [Interculturalism. The Quebec perspective].

[CR8] Cantle T (2008). Community cohesion.

[CR9] Caponio T, Caponio T, Borkert M (2010). Conclusion. Making Sense of Local Migration Policy Arenas. The Local Dimension of Migration Policymaking.

[CR10] Caponio T, Jubany O, Guel B (2016). Civic Integration Policies from Below: accounting for processes of convergence and divergence in four European cities, with Olga Jubany and Berta Güell. Ethnic and Racial Studies.

[CR11] Caponio T, Ricucci R, Zapata-Barrero R (2015). Interculturalism: a policy instrument supporting social inclusion?. Interculturalism in Cities.

[CR12] Casa del Quartiere di Torino, C. (2012). *Manifesto delle Case del Quartiere [Neighbourhood Houses' Manifesto]* http://www.casedelquartieretorino.org/manifesto-delle-case-del-quartiere-di-torino/. Accessed 25 July 2017

[CR13] Dekker R, Emilsson H, Krieger B, Scholten P (2015). A local dimension of integration policies? A comparative study of Berlin, Malmö, and Rotterdam. International Migration Review.

[CR14] Joppke C (2007). Beyond national models: Civic integration policies for immigrants in Western Europe. West European Politics.

[CR15] Lipsky M (1980). Street-level bureaucracy. Dilemmas of the individual in public services.

[CR16] Lüken-Klaßen D, Heckmann F (2010). Intercultural policies in European cities.

[CR17] Meer N, Modood T, Zapata-Barrero R (2016). Multiculturalism and Interculturalism: Debating the dividing lines.

[CR18] Penninx R, Martiniello M, Penninx R, Kraal K, Martiniello M, Vertovec S (2004). Integration policies and processes: State of the art and lessons. Citizenship in European cities. Immigrants, local politics and integration policies.

[CR19] Rocher F, Zapata-Barrero R (2015). Interculturalism in Montreal and Barcelona. Interculturalism in cities. Concept, policy and implementation.

[CR20] Roman, E. (2014). Neighbourhood houses. Case del Quartiere, Torino (Italy), EU-MIA (European migrant integration academy) research project, http://www.eu-mia.eu/media/library/20-01-2014-15-00-59. Accessed 25 July 2017

[CR21] Salée D (2010). Penser l’aménagement de la diversité ethnoculturelle au Québec: Mythes, limites et possibles de l’interculturalisme [Thinking of managing intercultural diversity in Quebec. Myths, limits and potentialities of interculturalism]. Politiques et Société.

[CR22] Schiller, M. (2015). Paradigmatic pragmatism and the politics of diversity. *Ethnic and Racial Studies.* doi:10.1080/01419870.2014.992925

[CR23] Scholten P (2013). Agenda dynamics and the multi-level governance of migrant integration. The case of Dutch migrant integration policy. Policy Sciences.

[CR24] Scholten, P., Collet, E., & Petrovic, M. (2017). Mainstreaming migrant integration? A critical analysis of a new trend in integration governance. *International Review of Administrative Sciences, 83*(2). doi:10.1177/0020852315612902

[CR25] Schön DA, Rein M (1994). Frame reflection: Toward the resolution of intractable policy controversies.

[CR26] Uberoi V, Modood T (2015). Multiculturalism rethought. Interpretations, dilemmas and new directions.

[CR27] Vermeulen F, Stoijtin R, Caponio T, Borkert M (2010). Local policies concerning unemployment among immigrant youth in Amsterdam and in Berlin: Towards strategic replacement and pragmatic accommodation. The local dimension of migration policymaking.

[CR28] Vertovec S, Wessendorf S (2010). The multiculturalism backlash: European discourses and practices.

[CR29] Zapata-Barrero R (2016). Exploring the foundations of the intercultural policy paradigm: A comprehensive approach. Identities: Global Studies in Culture and Power.

